# Feasibility of a combined intermittent theta-burst stimulation and video game-based dexterity training in Parkinson’s disease

**DOI:** 10.1186/s12984-023-01123-w

**Published:** 2023-01-12

**Authors:** Manuela Pastore-Wapp, Brigitte C. Kaufmann, Thomas Nyffeler, Simona Wapp, Stephan Bohlhalter, Tim Vanbellingen

**Affiliations:** 1 Neurocenter, Luzerner Kantonsspital, Lucerne, Switzerland; 2 ARTORG Center for Biomedical Engineering Research, Gerontechnology and Rehabilitation Group, University of Bern, Bern, Switzerland; 3 Sorbonne Université, Institut du Cerveau – Paris Brain Institute – ICM, Inserm, CNRS, Paris, France; 4 Department of Neurology, Inselspital, University Hospital, University of Bern, Bern, Switzerland; 5 Department of Neurology, University of Zurich, Zurich, Switzerland

**Keywords:** Parkinson’s disease, Dexterity, Video game-based training, Intermittent theta-burst stimulation, Feasibility

## Abstract

**Background:**

Persons with Parkinson’s disease (PD) often exhibit difficulties with dexterity during the performance of activities of daily living (ADL), inter alia due to dysfunctional supplementary motor area (SMA). Combined intermittent theta-burst stimulation (iTBS) over the SMA followed by video game-based training (VBT) may therefore improve dexterity related ADL. The VBT may induce high flow levels related to high performance during the training. The aim of this study is to evaluate the feasibility of a combined iTBS-VBT intervention in persons with PD.

**Methods:**

A total of nine persons with PD (mean age 63.3 ± 8.76 years) with self-reported difficulties with dexterity related ADL were included in this pilot iTBS-VBT study. All participants received either iTBS or sham stimulation over the SMA followed by a 45-min VBT, three times a week for a total of three weeks. Feasibility was measured by means of the adherence rate and the system usability (System Usability Scale). Moreover, flow was measured after the last VBT session.

**Results:**

Adherence rate was excellent with 100%. High system usability scores (i.e., mean 80%, range 55–97.5) and a significant Spearman’s correlation with the Flow State Scale (r = .762, p = .017) further point to the high feasibility of the VBT. Neither demographic variables nor difficulties in dexterity related ADL affected the usability of the VBT.

**Conclusion:**

This study demonstrates the high feasibility of a combined iTBS-VBT intervention. Moreover, the level of self-reported usability was related to flow experience. Whether this kind of combined iTBS-VBT intervention improves dexterity will be evaluated in a randomized controlled trial.

*Trial registration* clincaltrials.gov NCT04699149, date of registration 1. June 2021

## Background

Parkinson’s Disease (PD) is one of the most common movement disorders and represents a growing neurodegenerative condition [1]. Persons with PD often exhibit dexterity-related difficulties, both in performing basic (grooming, fastening buttons) and instrumental activities (cooking, writing, organizing pills in a pill holder) of daily living (ADL) [2–5]. These difficulties increase the burden of the disease and reduce health-related quality of life.

Physical training (e.g. strengthening training) and activity training is known to improve upper limb functions of persons with PD [6]. Ideally, the training creates a so-called flow state in which attention becomes “an experience in itself”. Flow is a subjective psychological state in which one performs best and forgets time and the surrounding other than the activity itself. [7, 8]. High flow levels are related to increased performance in any sort of task and at the state of optimal performance denoted by smooth and accurate performance [9]. Exergames, respectively video game-based training (VBT), aim to combine physical training and motivational aspects. Therefore, it is highly desirable to get into a flow in VBT.

Exergaming has been rapidly developing in PD neurorehabilitation and has been proven to be feasible and safe, showing already some promising results in improving different motor outcomes, such as gait and balance [10–12]. So far, a few studies showed good feasibility of exergaming based dexterity training in PD, and some potential for short-term efficacy improving dexterity [13, 14]. However, no studies were able to show long-term effects on dexterity, and the devices used so far, such as the Leap Motion Controller, LMC™ [13] only focused on visual and or verbal feedback during exergaming.

It is well known that persons with PD may demonstrate weaker [15] and less precise grip [16], which consequently may contribute to disturbed performance in dexterity related ADL [2]. So, adding tactile pressure feedback could further trigger motor learning during exergaming, as suggested before [17]. A recently developed device, called GripAble [18] may provide this kind of feedback. The GripAble is an exergaming device consisting of a handheld flexible sensor system. It is able to reliably detect both grip force and six degrees-of-freedom acceleration, enabling training of precision grip control, finger extension and wrist movements [19]. A multimodal VBT intervention using two types of devices (GripAble and LMC™) has not been done before to train dexterity in PD.

To further boost the effects on dexterity, the VBT sessions were combined with a facilitatory intermittent theta-burst protocol (iTBS) [20, 21], a type of repetitive transcranial magnetic stimulation, which is expected to produce behavioral effects not only outlasting the single administration (short term), but also retained longer after multiple applications [22]. iTBS over supplementary motor area (SMA), a cortical region being involved in fine motor control [23, 24], may bring the brain in an optimal state to facilitate subsequent training effects [25].

The aim of the present pilot study was to evaluate the adherence and usability of the combined iTBS-VBT intervention in persons with PD. The question was, if participating in this iTBS-VBT study was doable, although having to come over nine times over three weeks at an outpatient clinic and if the devices for the VBT were usable for persons with PD. Moreover, the flow experience during the VBT and a possible relation to usability was evaluated. We hypothesized that the combined iTBS-VBT intervention is feasible for persons with PD. Furthermore, we expected a relationship between usability and flow experience.

## Methods

### Aim

The main aim was to extensively evaluate the feasibility of the study design of the combined iTBS-VBT intervention and the flow experience during the VBT in persons with PD. The second aim was to assess the adherence to the protocol. And the third aim was to investigate whether demographic variables (i.e., age, disease duration, cognition, dexterity related ADL in PD) could interfere with the feasibility of the VBT.

### Design

In this pilot study of a randomized controlled trial, all nine participants received 45-min manual dexterity intervention three times a week for a period of three weeks after a baseline assessment (hereinafter referred to as T0). For the study protocol of the randomized controlled trial please see Pastore-Wapp et al. [26]. In short, the participants in the experimental group received nine VBT sessions each time preceded by true iTBS stimulation over the SMA and the participants in the control group received VBT with a preceding sham TMS each time. Participants were naïve to the TMS (iTBS or sham).

Follow-up measurement (T1) were carried out after a period of three weeks. The study was approved by the Ethics Committee for Northwest and Central Switzerland (EKNZ), Switzerland (number 2019–00433) and was conducted in accordance with the Helsinki Declaration and the Guidelines of Good Clinical Practice. Trial registration was done on clinicaltrials.gov with the identification code: NCT04699149.

### Participants

Nine persons with PD were recruited at the Neurocenter, Lucerne Cantonal Hospital, Switzerland. Informed consent was signed prior to subject enrolment. For more detail of the study protocol see Pastore-Wapp et al. [26]. Participants were included if they met the following criteria: (I) confirmed PD according to the UK Parkinson’s Disease Society brain bank criteria [27], (II) experiencing subjective dexterous difficulties in performing ADL, (III) aged 18–90 years, (IV) ability to understand and to follow the study procedures, (V) no severe cognitive impairment according to the Montreal Cognitive Assessment (MoCA) (scores < 21), (VI) no other neurological, psychiatric, or developmental diseases prior to PD diagnosis. Exclusion criteria for iTBS use were current pregnancy, personal history of epilepsy or seizures, cardiac pacemaker or brain metal implants [28].

### Material

At T0 handedness [29], time since diagnosis, Hoehn and Yahr (H & Y) Staging Scale [30] and the MoCA [31] were assessed.

At T1 participants filled out a modified version of the well validated Flow State Scale for Occupational Therapy (FSSOT) [32], which consists of 11 items. For example, “I lost track of time while doing the task” or “I really enjoyed what I was doing”. Items of the FSSOT were scored from 1 (strongly disagree) to 5 (strongly agree), resulting in a maximum level of 55 where higher scores represent higher flow experience.

Also, the System Usability Scale (SUS), which is a well-validated questionnaire for usability evaluation (satisfaction, efficiency), consisting of a 10-item, 5-point Likert scale was filled out at T1. Each item was scored from 1 (strongly disagree) to 5 (strongly agree). The final SUS score range from 0 to 100% where higher score indicates better system usability. Scores of 70% and higher represents acceptable to excellent usability [33, 34].

Additionally, adherence rate was determined as the ratio of the number of sessions performed (SP) and the planned number of sessions (PS), which is 9: SP/PS × 100% = SP/9 × 100%. An adherence rate of 80% or higher was considered as good.

To evaluate dexterity related ADL in PD the Dexterity Questionnaire 24 (DextQ-24) was assessed at T0 which is a standardized patient self-rated outcome measure [3]. This questionnaire contains 24 questions, which are divided into five subgroups (“washing/grooming”; “dressing”; “meals and kitchen”; “everyday tasks”; “TV/CD/DVD”). Score ranges from a minimum of 24 to a maximum of 96 points. Higher values correspond to more difficulties in dexterity related ADL.

#### Intermittent theta-burst stimulation

A MagPro R30 stimulator (Medtronic Functional Diagnostics, Skovlunde, Denmark) with a figure-of-eight coil (Magnetic Coil Transducer MC-B70, Medtronic) or a similar looking sham coil (Magnetic Coil TransducerMC-P-B70, Medtronic) over the SMA was used. The same iTBS protocol described by Huang et al. (2005) was applied [35]. In brief, bursts of 3 pulses delivered at 50 Hz were organized in stimulation trains. Every stimulation train contained 10 bursts, interspaced by 200 ms, thus lasting 2 s. The stimulation train was repeated 20 times, in blocks of 10 s (i.e., a stimulation train of 2 s and a break of 8 s). This resulted in a total of 600 pulses. Sham stimulation was applied by the same iTBS protocol using the sham coil. Immediately after stimulation they started with the VBT with the more affected hand.

#### Video game-based training (VBT)

For the VBT two different devices were used: (I) The LMC™ (https://www.leapmotion.com/) which is an optoelectronic commercially available device suitable for hand gesture-controlled user interfaces allowing human–computer interaction and (II) GripAble (https://gripable.co/), a device developed for hand function problems. Each device was used for about 15 min.

LMC™ tracks hand and finger movements by modelling a physiological hand and finger joints within a virtual reality (VR) environment [36, 37]. The patients see their hand in real time on the screen and can then played different games. Games are described in detail elsewhere (see Pastore-Wapp et al. [26]). In short GripAble can be connected to a tablet on which a GripAble app including different therapy games. It allows the training of upper-arm and hand movements during wrist extension and flexion, pronation and supination, wrist radial and ulnar deviation as well as hand/finger grip-force [38]. From the nine available games, five were chosen to be used, since each of these five games focus on different hand/finger movements.

### Statistical analysis

Descriptive statistics were used to present baseline characteristics and results of outcome measurements. To explore relationships between clinical parameters, usability scores and flow scores respectively, Spearman’s correlations were performed with a level of significance at p < 0.05 (two-tailed). All analyses were performed using IBM28 (IBM Corp. Released 2021. IBM SPSS Statistics for Windows, Version 28.0. Armonk, NY: IBM Corp).

## Results

Recruitment took place between November 2020 and May 2022. A total of nine participants started and completed the intervention. Participants were aged 46–79 years (mean 63.33 ± 8.76, 5 female) with H & Y stages I to III [30]. 44.4% received iTBS, 55.6% sham stimulation. For more detail about clinical and demographic characteristics see Table 1.

**Table 1:** Clinical and demographic characteristics

Characteristic	
Age in years, mean (SD)	63.33 (8.76)
Gender, n (%)	
Male Female	4 (44.4) 5 (55.6)
Handedness, n (%)	
Right Left Ambidexter	8 (88.9) 0 (0) 1(11.1)
H & Y	
I II III	3 (33.3) 3 (33.3) 3 (33.3)
Disease duration in months, mean (SD) Range in months	86.56 (87.37) 10–261
DextQ-24	34.78 (4.12)
Cognitive function MoCA (max 31), mean (SD)	27.89 (2.21)

Estimated means with standard deviations (SD) or absolute values with percentage (%) are presented, *H & Y* Hoehn and Yahr stages, *DextQ-24* Dexterity Questionnaire 24, *MoCA* Montreal Cognitive Assessment

Adherence rate was excellent with 100%. Every participant completed every planned session. No adverse events related to the iTBS or dropouts were reported.

Regarding usability of the VBT the mean SUS score was 80 (range 55–97.5), indicating very good usability of the used training devices. Only one of the nine participants rated the system with 55%, still meaning acceptable usability. Flow was rated from 38 to 52 (mean 44.89), which is very high. There was a significant correlation between flow and SUS with r = 0.762, p = 0.017.

There were no significant correlations between SUS and age (r = 0.107, p = 0.784), disease duration (r = − 0.159, p = 0.683), MoCA (r = − 0.193, p = 0.618), and DextQ24 (r = 0.047, p = 0.904).

## Discussion

This pilot study evaluated the feasibility of the study design, measured by means of usability and adherence, of a 3-week combined iTBS-VBT intervention in PD. The exceptional adherence rate of 100% to the training protocol points to the excellent feasibility of the combined intervention. The iTBS-VBT intervention was appreciated by the participants which resulted in high overall usability and flow experience. The latter experience points out that the participants were well engaged during training. Higher usability was also related with better flow experience. Demographic factors (i.e. age, disease duration, cognition, and dexterity related ADL in PD) did not affect the usability of the multimodal iTBS-VBT intervention.

The excellent feasibility may have several reasons. First, participating in a 3-week intervention is doable for most persons with PD, although having to come over three times a week at the outpatient clinic. Longer outpatient trials often suffer from less adherence due to early drop-outs since patients or their caregiver may experience longer trials as a burden [39]. Lack of motivation to continue long duration training has also been mentioned [40]. Second, the attractive training modality of VBT certainly may have played a role as well, as previous studies already showed for VBT in PD [13, 14, 41]. Third, impaired dexterity is an often-mentioned problem in PD [2, 3, 42, 43] and still neglected in PD rehabilitation research, shown by the fact of very few ongoing randomized controlled trials worldwide (clinicaltrials.gov). The participants were therefore motivated to participate and conclude this trial. Finally, the iTBS application was well tolerated by the participants, which is in line with the excellent tolerability of TBS recently reported by a systematic review [44]. Also, the short stimulation duration of iTBS, and the lower stimulation pulse intensity as compared to traditional repetitive transcranial magnetic stimulation protocols may have further contributed to the high feasibility of the training.

GripAble and the LMC™ were new to all participants in our study, and most of them picked up the use very quickly. They appreciated the easy integration of the different functions in the two systems, wanted to use the devices again and felt very safe using them. The high applicability of this multimodal VBT intervention also speaks for the possibility of using VBT not only in the outpatient setting, but also as a home-based training. However, this will need to be evaluated in future studies.

The participants experienced high flow during the VBT sessions, suggesting that they were well involved and found the game setting to be very motivating. Both devices had challenging games with different levels of difficulty and built-in feedback to optimize performance, so there was no routine or boredom, which in turn can negatively influence the flow experience. High flow levels are known to increase performance [9] and motivation levels [8]. Therefore, high flow levels during the training are very desirable. Interestingly, both usability and flow were significantly associated which means that the better a system was accepted by its users the better one got into a flow. This is in accordance with other virtual reality and VBT interventions, which also showed enhanced motivation during training, and high adherence rates [12, 14, 45]. The high system usability also suggest that the devices were useful to help guiding persons with PD to their own challengeable objectives, which is very important to get into a flow. This integrative model of usability and flow may further explain the participants high satisfaction as well [46], which then resulted in an optimal gaming experience.

We found no associations between demographic factors and usability scores, supporting the generalizability of the combined iTBS-VBT intervention, and suggesting that the intervention may be appropriate for a wide range of persons with PD. However, one has to be careful with this assumption since we only included persons with relatively intact cognitive function and mild to moderate PD.

## Conclusion

This study demonstrates high feasibility of a combined multimodal iTBS-VBT intervention in PD. Moreover, during the VBT, the level of self-reported usability seems to be related to the level of flow experience. Whether and how long lasting this kind of combined iTBS-VBT intervention improves dexterity will need to be evaluated in a randomized controlled trial.

## Data Availability

Anonymized raw data are available and can be shared upon appropriate request.

## Abbreviations

ADL: Activities of daily living
DextQ-24: Dexterity Questionnaire 24
FSSOT: Flow State Scale for Occupational Therapy
H & Y: Hoehn and Yahr stages
iTBS: Intermittent theta-burst stimulation
LMC™: Leap motion controller ™
MoCA: Montreal Cognitive Assessment
PD: Parkinson’s disease
SMA: Supplementary motor area
SUS: System Usability Scale
VBT: Video game-based training
